# Parliament and the Profession

**Published:** 1919-03-15

**Authors:** 


### PARLIAMENT AND THE PROFESSION.

### Tuberculous Ex-Soldiers

Major Astor stated that a conference was being held in order to encourage, under suitable conditions and on approved standards of efficient working, centres or colonies where the tuberculous can follow the occupation of agriculture or gardening, or any form of employment to which they are adapted.

### Patent Medicines.

Major Astor, speaking for the Home Secretary, informed Sir H. Norman that the Home Office had under consideration the whole question of the increase of advertisements of patent and proprietary medicines of the classes described as contrary to the public interest by the Report of the Select Committee on Patent Medicines, 1914.

### Shortage of Dentists.

Dr. Addison informed Commander Bellairs that there is no doubt that a serious shortage of qualified dentists prevails in this country. The report of the Departmental Committee charged with the subject was laid before Parliament last week with a view to its circulation at an early date, when the Government will be in a position to approach the whole problem with the best information available.

### Navy and Army Masseuses.

Captain Guest informed Mr. Rawlinson that there are no uncertificated masseuses in the employ of the War Office. Asked as to the reason for the difference in rates paid to masseuses in the Army and the Navy, Captain Guest stated that the new rates recently introduced were adopted after very full consideration of the rates paid by other departments and by civil employers. Certain representations regarding them are, however, under consideration.

### Military Occupation of Mental Hospitals.

Sir Cyril Cobb asked the Secretary for War as to the continued use of the Horton Hospital, the Manor Hospital, and the Ewell Colony, and was informed by Captain Guest that as sick from France were still arriving at the rate of 1,000 per day, accommodation was still needed. The Manor Hospital, however, would be closed from March 15 next.

